# Characterization of the Mitochondrial Genome Landscape in MSCs, iPSCs and iMSCs from Osteoarthritis Patients and Healthy Donors

**DOI:** 10.3390/genes17091101

**Published:** 2026-09-11

**Authors:** Vasileios Konteles, Ioanna Papathanasiou, Maria Tzetis, Eugenios Goussetis, Kostantinos Malizos, Aspasia Tsezou

**Affiliations:** 1 Laboratory of Cytogenetics and Molecular Genetics, Faculty of Medicine, University of Thessaly, 41110 Larissa, Greece; kontelesb@gmail.com (V.K.); iopapat@uth.gr (I.P.); 2 Department of Medical Genetics, Medical School, National and Kapodistrian University of Athens, 11527 Athens, Greece; mtzetis@med.uoa.gr; 3 Stem Cell Transplant Unit, Aghia Sophia Children’s Hospital, 11527 Athens, Greece; evgousetis@gmail.com; 4 Department of Orthopaedics, Faculty of Medicine, University of Thessaly, 41110 Larissa, Greece; knmalizos@gmail.com

**Keywords:** iMSCs, mitochondrial SNVs, transcriptome, ncRNAs, osteoarthritis

## Abstract

**Background/Objectives:** Osteoarthritis (OA) is strongly associated with mitochondrial dysfunction, oxidative stress, and the accumulation of mitochondrial DNA (mtDNA) alterations in several joint-associated cell types, including chondrocytes, synoviocytes, and mesenchymal stromal cells (MSCs). These alterations contribute to impaired cellular homeostasis and reduced regenerative potential. In this study, we systematically characterized mtDNA heteroplasmy and variant distribution across bone marrow MSCs, induced pluripotent stem cells (iPSCs) and induced MSCs (iMSCs) derived from OA patients and healthy donors. **Methods:** Ultra-deep mitochondrial genome sequencing was integrated with transcriptomic and miRNome analyses to investigate mitochondrial remodeling during cellular reprogramming, encompassing changes in mtDNA heteroplasmy, variant distribution, mtDNA copy number and associated transcriptomic adaptations of nuclear-encoded mitochondrial pathways. **Results:** OA-derived MSCs exhibited a markedly increased heteroplasmic burden, followed by decrease in mtDNA copy number and accumulation of non-synonymous variants, particularly within OXPHOS-related genes, including MT-ND1-5, MT-ATP8, MT-CO1, and MT-RNR1/2. Reprogramming into iPSCs and subsequent differentiation into iMSCs were associated with an increase in mtDNA copy number and a progressive reduction in heteroplasmic variants predicted to have pathogenic potential, including m.7913C>T and m.7821G>A, as well as substantial reduction in heteroplasmic variant burden within several mitochondrial genes, suggesting mitochondrial genome remodeling during cellular reprogramming. Multiomic analysis further revealed coordinated deregulation of mitochondrial-associated nuclear genes and ceRNA regulatory networks involving lncRNAs MEG3 and SNHG14, along with multiple mitochondria-related miRNAs. These findings suggest post-transcriptional regulations associated with mitochondrial adaptation in iMSCs **Conclusion:** Collectively, our findings suggest that cellular reprogramming is associated with mitochondrial genomic reorganization in the donor-matched cell populations examined. These exploratory observations support the utility of iMSCs as a relevant model for studying OA-associated mitochondrial alterations and as a potential tool for regenerative approaches in OA.

## 1. Introduction

Osteoarthritis (OA), the most prevalent joint disease worldwide, is predominantly marked by synovial inflammation, subchondral bone remodeling and cartilage matrix degradation. Among OA risk factors, age over 50 years has been closely linked to OA pathogenesis contributing to inflammaging, cellular senescence, oxidative stress and impaired mitochondrial function [1]. As mitochondrial dysfunction is a hallmark of aging, the role of mitochondria in OA has attracted increased attention [2,3].

Mitochondria are multifunctional organelles that maintain cellular homeostasis by integrating bioenergetic processes with redox regulation and broader metabolic networks [4]. In addition to their dependence on nuclear-encoded proteins, mitochondria harbor their own (~16.5 kb) circular genome, rendering mitochondrial function on tight coordination between nuclear and mitochondrial genetic systems. Importantly, the functional consequences of heteroplasmic mutations are typically threshold-dependent with biochemical and cellular defects becoming evident only when mutant mtDNA exceeds a critical level [5]. In addition to heteroplasmy, mitochondrial DNA (mtDNA) copy number represents an important feature of mitochondrial biology and is dynamically regulated during cellular reprogramming and differentiation. These changes reflect the extensive mitochondrial reorganization that accompanies transitions in cellular identity [6].

In OA, mitochondrial dysfunction has been linked to alterations in the mitochondrial genome itself. Increased mtDNA damage, accumulation of somatic mtDNA variants and elevated heteroplasmy levels have been reported in OA-associated cell populations, such as chondrocytes and synoviocytes [3,7] suggesting that mtDNA instability may actively contribute to disease development and progression. Furthermore, oxidative-stress-induced mtDNA alterations together with limited mtDNA repair capacity and clonal expansion of deleterious variants [8] are thought to exacerbate mitochondrial dysfunction, thereby establishing a pattern that promotes cartilage degeneration and impaired joint homeostasis.

In addition to chondrocytes, other cells within the OA joint, such as aged mesenchymal stromal cells (MSCs), display altered energy production, reduced oxidative phosphorylation efficiency and increased production of reactive oxygen species (ROS), contributing to cellular stress, phenotypic instability and reduced regenerative capacity [9]. Increasing evidence supports that regulation of mitochondria dynamics and function is imperative for MSCs differentiation. Cell fate transitions, such as somatic cell reprogramming, are accompanied by extensive metabolic and mitochondrial remodeling. During conversion of MSCs into induced pluripotent stem cells (iPSCs), a pronounced metabolic shift from oxidative phosphorylation toward glycolysis has been consistently reported, reflecting the bioenergetic requirements of pluripotency [10,11]. Redifferentiation of iPSCs into induced mesenchymal stromal cells (iMSCs) partially restores the mitochondrial phenotypic characteristics of primary MSCs. Although iMSCs regain aspects of mesenchymal stem cells’ identity and increase their reliance on oxidative metabolism, several studies have reported persistent differences in mitochondrial structure [12], bioenergetic function [13] and metabolic flexibility when compared to tissue-derived MSCs. Moreover, reprogramming to pluripotency has been associated with substantial changes in mtDNA variant composition and heteroplasmy levels, including the selective removal of deleterious mtDNA variants through purifying selection, stochastic enrichment of specific variants through clonal selection, and the emergence or expansion of new mtDNA variants [10]. Changes in mtDNA copy number represent an additional aspect of mitochondrial remodeling during reprogramming, although the direction and magnitude of these changes appear to depend on the cellular and metabolic context [14,15]. Increased mtDNA copy number has been reported in specific reprogramming conditions and may accompany enhanced mitochondrial biogenesis, while differentiation of iPSCs can also promote mtDNA expansion together with increased mitochondrial respiratory activity [15,16]. Cellular reprogramming is also associated with profound changes in mitochondrial dynamics, including fusion, fission, and mitophagy, which contribute to mitochondrial quality control and adaptation during changes in cell fate [17,18]. The above observations support the existence of mtDNA selection mechanisms operating during cellular reprogramming [19], which may act to favor mitochondrial genomes compatible with the pluripotent state.

Until now, most studies in OA cell populations have primarily examined the global mitochondrial mutation burden without resolving how mtDNA variants are distributed across individual mitochondrial genes or respiratory chain complexes [20], or whether cellular reprogramming effectively resets OA-associated mitochondrial genomic alterations [10,21].

In the present study, we systematically characterize mtDNA heteroplasmy, variant distribution and mtDNA copy number across donor-matched bone-marrow-derived-MSCs, iPSCs and iMSCs obtained from three OA patients and two young healthy donors. By integrating deep mitochondrial genome sequencing with transcriptomic and miRNome data, we aim to describe mitochondrial genomic changes across the MSC, iPSC and iMSC trajectory and to explore their association with the expression of nuclear-encoded mitochondrial genes. The study was designed to provide a comprehensive descriptive analysis of mitochondrial genome remodeling rather than to directly investigate the mechanisms underlying mitochondrial rejuvenation during cellular reprogramming.

## 2. Materials and Methods

### 2.1. Bone Marrow Samples

The study was designed as an exploratory, donor-matched analysis in which cell populations derived from each donor were evaluated longitudinally across the MSC, iPSC and iMSC cellular stage. Bone marrow samples were collected from 3 osteoarthritis (OA) patients and 2 healthy donors. The OA cohort comprised two females (69 years old, K/L = 3, BMI = 28.3 and 76 years old, K/L = 4, BMI = 29.1) and one 79-year-old male (K/L = 4, BMI = 25.7). Bone marrow aspirates were obtained from the proximal metaphysis of the femur immediately following femoral neck osteotomy during total hip arthroplasty at the Orthopaedics Department of the University Hospital of Larissa, Greece. Healthy donors, one female and one male, ages 6 and 13 years, were pediatric cell-transplant volunteers at the Bone Marrow Transplantation Unit (BMTU) of “Aghia Sophia” Children’s Hospital in Athens, Greece. Bone marrow aspirates were collected from the medial iliac crest and were available at the BMTU. The study protocol was approved by the Ethics Committees of both “Aghia Sophia” Children’s Hospital and University Hospital of Larissa and complied with the principles of the Declaration of Helsinki (1975). Informed consent was obtained from all OA patients and from the parents or legal guardians of the underaged healthy donors.

### 2.2. BM-MSCs Isolation, iPSC Generation and Differentiation into iMSCs

Isolation of BM-MSCs (passage 1–6), reprogramming into iPSCs (passage 5–20), and subsequent differentiation into induced MSCs (iMSCs) (passage 4–10) followed established protocols previously described by us [22,23]. Briefly, BM-MSCs from OA patients (MSC_OA) and healthy donors (MSC_N) were expanded in DMEM supplemented with 10% FBS, 1% Pen/Strep, HEPES, L-glutamine, ascorbic acid, sodium pyruvate and L-glucose under standard incubation conditions (37 °C, 5% CO_2_). Adherent mesenchymal-like cells appeared within the first days of culture and were subcultured upon confluence using trypsin at a 1:3 split ratio. Reprogramming of MSCs into iPSCs was performed using a modified synthetic mRNA-based approach, as previously reported [22]. In brief, MSCs were seeded onto irradiated NuFF feeder cells and repeatedly transfected with the five canonical reprogramming factors (OCT4, SOX2, KLF4, c-MYC and LIN28) in the presence of B18R. Emerging colonies (a single clone for each iPSC donor) with pluripotent morphology were manually selected and expanded in mTeSR1 medium on Matrigel-coated surfaces. For mesenchymal induction, iPSCs were subjected to embryoid body formation followed by adherence to gelatin-coated flasks in MSC differentiation medium, as previously described [22,23]. Resulting induced mesenchymal stem cells from OA patient (iMSC_OA) and healthy donor (iMSC_N) lines were subsequently expanded under the same culture conditions used for primary MSCs. Characterization of iMSCs included flow cytometric profiling of canonical MSC markers (CD90, CD44, CD105, CD73, CD29) and exclusion of hematopoietic markers (CD34, HLA-DR) [22,23]. Cell counts and viability were assessed using 7-aminoactinomycin D (7-AAD). Trilineage differentiation capacity (osteogenic, adipogenic and chondrogenic) was confirmed according to the manufacturers’ protocols using lineage-specific differentiation media followed by histochemical staining [22].

### 2.3. DNA Extraction and Mitochondrial DNA Amplification

Genomic DNA was extracted from MSC_OA (*n* = 3), MSC_N (*n* = 2), iPSC_OA (*n* = 3), iPSC_N (*n* = 2), iMSC_OA (*n* = 3) and iMSC_N (*n* = 2) samples using a Qiagen DNA extraction kit (Qiagen, Hilden, Germany) according to the manufacturer’s instructions. DNA concentration and purity were assessed using a NanoDrop 2000 spectrophotometer (mean A260/A280 value = 1.86) (Thermo Fisher Scientific, Waltham, MA, USA) and a Qubit 4.0 fluorometer (Thermo Fisher, Waltham, MA, USA), while genomic integrity was evaluated by electrophoresis on 1% agarose gels. To enrich and linearize the mitochondrial genome, long-range PCR was performed to amplify two overlapping fragments spanning the entire mitochondrial sequence (rCRS, GenBank ID NC_012920). Amplicons were generated using primer sets previously described [24]: Set 1 (5042f–1424r, 12.96 kb) and Set 2 (528f–5789r, 8.7 kb). Primer specificity was validated by the absence of amplification in Rho0 control DNA, confirming that nuclear mitochondrial pseudogenes (NUMTs) were not co-amplified. PCR reactions were performed using LongAmp Taq DNA Polymerase (New England Biolabs, Ipswich, MA, USA) in 25 μL reaction volumes containing 50 ng of genomic DNA. Amplified products were assessed on 0.8% agarose gels alongside positive and negative controls and subsequently quantified using a Qubit 4.0 fluorometer. Purification of PCR products was carried out using AMPure XP beads (Beckman Coulter, Brea, CA, USA). Following purification, amplicons were pooled in defined equimolar ratios (0.35 × Set 2 and 0.65 × Set 1) to ensure proportional representation of each fragment. The pooled mixture was requantified and subjected to a second purification step using the QIAquick PCR Purification Kit (Qiagen, Hilden, Germany) prior to downstream library preparation and sequencing.

### 2.4. Mitochondrial DNA Deep-Sequencing

Pooled amplicons were tagmented, amplified, purified, normalized and subsequently combined into a single Illumina library using the Nextera XT DNA Sample Preparation Kit (Illumina, San Diego, CA, USA), according to the manufacturer’s instructions. For library preparation, 1 ng of DNA per sample was used. Resulting libraries were quantified using Qubit 4.0 Fluorometer (Thermo Fisher, Waltham, MA, USA) and evaluated for fragment size distribution and overall quality using Agilent 2100 Bioanalyzer (Agilent Technologies, Santa Clara, CA, USA). Libraries were then normalized to equimolar concentrations and pooled into a multiplexed library suitable for high-throughput sequencing. Sequencing was performed on an Illumina MiSeq platform employing the MiSeq Reagent Kit v3.0 (Illumina, CA, USA) to generate paired-end reads of 2 × 150 bp. Sequencing runs were performed to a minimum depth of approximately 6000× per sample, enabling robust detection of low-frequency mitochondrial variants and accurate estimation of heteroplasmy levels. Across all samples, the mean mitochondrial mapping efficiency was 96%, the mean duplication rate was 12% and 89% of mtDNA-aligned reads were retained after quality filtering.

### 2.5. Variant Calling and Bioinformatic Analysis

Raw sequencing reads were subjected to quality control using FastQC (v0.11.8) and subsequently aligned to the human mitochondrial reference genome (NC_012920.1) using the bioinformatic tool BWA (v0.7.17). The resulting alignment files were sorted and indexed with Samtools (v1.12), and PCR duplicates were identified and marked with Picard (v2.2.4). Mitochondrial single nucleotide variants (mtSNVs) were detected using Mitoverse online platform (mtDNA-Server v2 Beta (rc12), v2.0.0), which incorporates Haplogrep and Haplocheck packages for variant calling, haplogroup assignment and evaluation of potential sample contamination. Heteroplasmy fraction (HF) was calculated, as the ratio of variant allele depth to reference allele depth. Variants with HF > 0.98 were classified as homoplasmic, whereas HF values of >0.02 and <0.98 were considered heteroplasmic. Heteroplasmic burden was defined as the total number of heteroplasmic variants detected per sample. To ensure high-confidence calls, we applied the following filtering criteria: (1) variant allele fraction (AF) > 2%; (2) mean mtDNA depth of coverage > 1500× to enable detection of low-level heteroplasmy; (3) retention of single-nucleotide variants only; (4) exclusion of variants exhibiting strand bias; and (5) removal of variants located in known low-complexity mtDNA regions (66–71 bp, 300–316 bp, 513–525 bp, 3106–3107 bp, 12,418–12,425 bp and 16,181–16,194 bp). To exclude sequencing-related artifacts, mean mitochondrial read depth was compared between OA and healthy donors. No statistically significant difference was detected (unpaired *t*-test), indicating that the observed heteroplasmy differences were unlikely to be driven by sequencing-depth variation. Putative pathogenicity of mtSNVs was evaluated using the MutPred algorithm, and variants scoring >0.60 were classified as potentially pathogenic in accordance with the MITOMAP Human Mitochondrial Genome Database (2026 Update #1).

### 2.6. mtDNA Copy Number Quantification

mtDNA copy number was assessed by quantitative real-time PCR (qPCR) using the mitochondrially encoded tRNA-Leu (UUR) 1 gene (MT-TL1) as the mitochondrial target and the nuclear β2-microglobulin (B2M) gene as the reference gene. qPCR amplification was performed on an ABI 7300 system (Applied Biosystems, Bedford, MA, USA). Primer sequences were as follows: B2M forward, 5′-TGCTGTCTCCATGTTTGATGTATCT-3′; B2M reverse, 5′-TCTCTGCTCCCCACCTCTAAGT-3′; MT-TL1 forward, 5′-CACCCAAGAACAGGGTTTGT-3′; and MT-TL1 reverse, 5′-TGGCCATGGGTATGTTGTTA-3′ [25,26]. Amplification was carried out using denaturation at 95 °C for 10 min, 40 cycles of 95 °C for 15 s and 60 °C for 1 min. Melting-curve analysis was subsequently performed to verify amplification specificity and confirm the presence of a single PCR product. All reactions were performed in duplicate. The mtDNA copy number was estimated from the difference between the Ct values of the nuclear and mitochondrial targets, and the estimated mtDNA copy number per diploid cell was calculated as mtDNA copy number = 2 × 2^ΔCt^.

### 2.7. Transcriptomic Data Analysis

Transcriptomic (mRNA and lncRNA) and miRNome datasets, previously generated in our laboratory [23], using identical biological samples, were reanalyzed for the purposes of this study. Differential expression analysis was performed on filtered datasets using DESeq2-normalized values [27]. The false discovery rate (FDR) was controlled using the Benjamini–Hochberg correction. Genes were considered differentially expressed when the adjusted *p*-value was ≤0.05 and the fold-change (FC) was >1.5 or <−1.5. Venn diagrams and heatmaps were generated using the R/Bioconductor packages VennDiagram and ComplexHeatmap, respectively. Nuclear-encoded mitochondrial genes were identified using the Human MitoCarta3.0 database [28]. Regulatory interactions between differentially expressed miRNAs and their predicted target genes and lncRNAs were visualized using the network package in R and the Cytoscape platform (v3.10.1).

### 2.8. Statistical Analysis

Statistical analyses were performed to evaluate the effects of donor status (OA vs. healthy) and cell type (MSCs, iPSCs, iMSCs) on mitochondrial variant burden and related parameters. For within-donor comparisons across the three cell states, non-parametric repeated-measures tests were used. Specifically, Friedman’s test was applied to compare total heteroplasmic variant counts and hotspot gene variant burden across MSCs, iPSCs and iMSCs derived from the same donors, followed by Wilcoxon signed-rank post hoc tests with Benjamini–Hochberg correction for multiple pairwise comparisons, where appropriate. For two-group comparisons between OA and healthy donors within the same cell type, the Mann–Whitney U-test was used. Mean mitochondrial sequencing depth was compared between OA and healthy samples using either the unpaired *t*-test or Mann–Whitney U-test, depending on the distribution of the data, to exclude depth-related bias in heteroplasmy estimates. Unless otherwise stated, statistical significance was defined as *p* < 0.05. All statistical analyses were performed in R/R studio (version v3.10) using main stats packages. Friedman’s tests were conducted using “friedman.test”, Wilcoxon and Mann–Whitney tests using “wilcox.test” and correlation analyses using “cor”. Graphs were generated in R (version 4.5.3) using “ggplot2” (version 4.0.3) while heatmaps and Venn diagrams were generated using “ComplexHeatmap” and “VennDiagram”, respectively. Network figures were prepared using Cytoscape (version 3.10.1).

## 3. Results

### 3.1. Reprogramming to iMSCs Reduces the Heteroplasmic Variant Burden of OA-Derived MSCs

**Figure 1:**
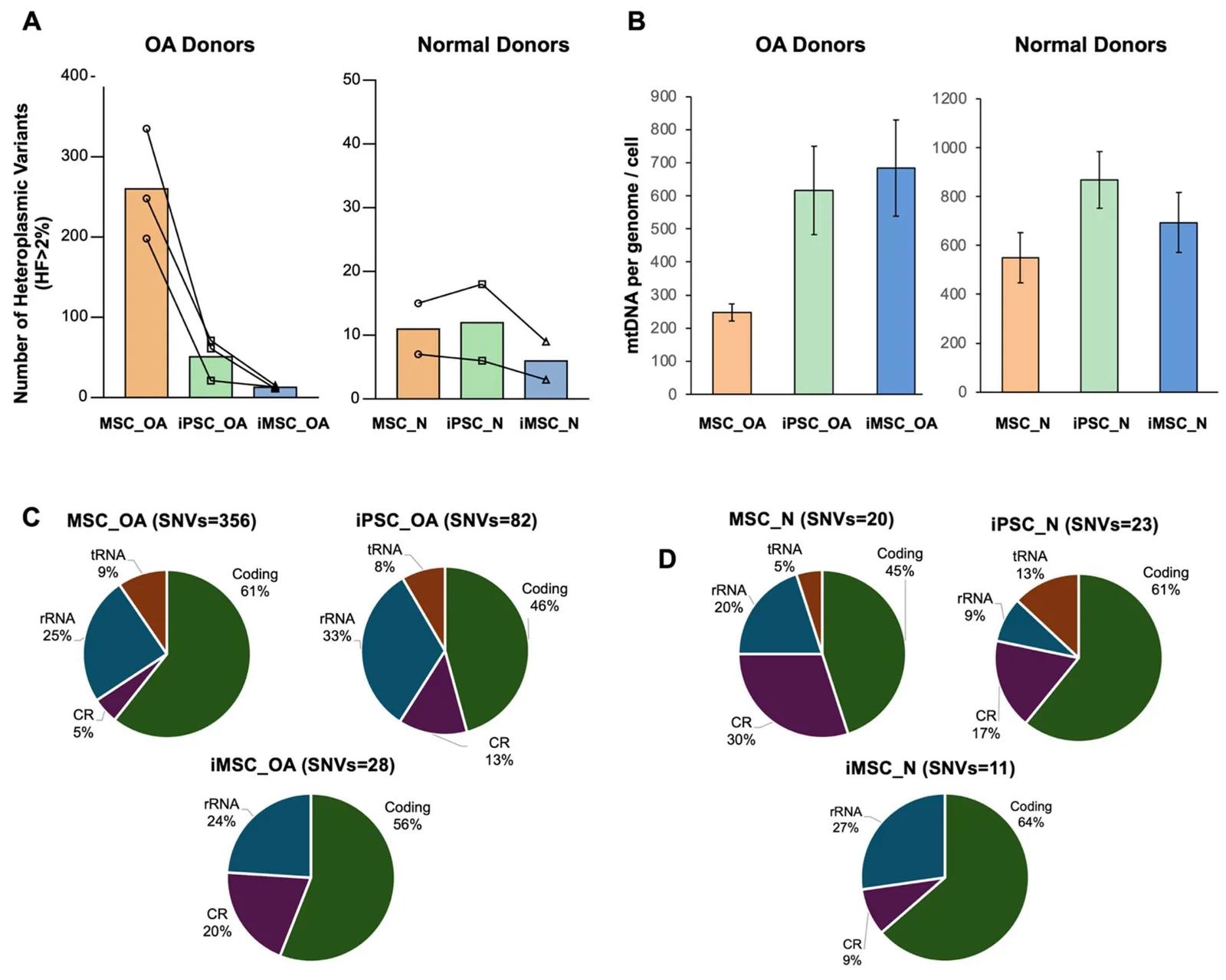
(**A**) Heteroplasmic SNVs burden (HF > 2%) across MSC_OA (MSC_OA1, MSC_OA2, MSC_OA3) (orange), iPSC_OA (iPSC_OA1, iPSC_OA2, iPSC_OA3) (green) and iMSC_OA (iMSC_OA1, iMSC_OA2, iMSC_OA3) (blue); and MSC_N (MSC_N1, MSC_N2) (orange), iPSC_N (iPSC_N1, iPSC_N2) (green) and iMSC_N (iMSC_N1, iMSC_N2) (blue). (**B**) mtDNA copy number in MSCs, iPSCs and iMSCs derived from OA and normal donors. Relative mtDNA copy number is expressed as the ratio of mitochondrial to nuclear DNA, using MT-TL1 as the mitochondrial target gene and B2M as the nuclear reference gene. All experiments were carried out in duplicate; error bars represent the SEM. (**C**,**D**) Pie charts showing the proportional distribution of variants across mitochondrial genomic regions (coding, control region (CR), tRNA, and rRNA) in OA- and healthy-derived cell types.

Quantitative assessment of mitochondrial single nucleotide variants (SNVs) across the studied donor cohort (MSCs, iPSCs, iMSCs) derived from osteoarthritis (OA) patients (*n* = 3) and healthy donors (*n* = 2) revealed clear differences in heteroplasmic variant burden among them. Specifically, we found that primary bone marrow MSC_OA exhibited markedly higher number of heteroplasmic variants (heteroplasmic fraction, HF > 2%) compared to MSC_N. On average, MSC_OA harbored 198–335 heteroplasmic SNVs per sample, whereas MSC_N displayed only 7–15 variants (Mann–Whitney U-test, *p* = 0.015, Figure 1A). Following cellular reprogramming and subsequent differentiation, progressive reduction in heteroplasmic load was detected in the OA group. Specifically, iPSC_OA showed a substantial decrease in the number of variants, while the corresponding iMSC_OA exhibited a slight further decline (Friedman’s test, *p* = 0.012, Figure 1A). Notably, within the OA cohort, inter-donor variability was evident primarily among MSCs (Friedman’s test, *p* = 0.042, Figure 1A), indicating that mitochondrial heterogeneity in OA may be donor-dependent. In contrast, in cell populations derived from healthy donors, the heteroplasmic load remained consistently low and stable across all cellular states (MSCs iPSCs, iMSCs), with no significant differences among them (Friedman’s test, *p* = 0.329) (Figure 1A). Given the inclusion of only two healthy donors, this pattern should be regarded as descriptive.

To further characterize the mitochondrial genome during cellular reprogramming, mtDNA copy number was relatively quantified. In OA-derived cells, mtDNA copy number increased from MSCs to iPSCs and remained elevated following differentiation into iMSCs, although these changes did not reach statistical significance (Friedman’s test, *p* = 0.058, Figure 1B). A similar increase was observed following reprogramming of MSCs from normal donors to iPSCs; however, mtDNA copy number was subsequently decreased in iMSCs, while remaining higher than primary normal MSCs (Friedman’s test, *p* = 0.411). Interestingly, despite the significantly lower mtDNA copy number observed in OA-MSCs compared with normal MSCs (Mann–Whitney U-test, *p* = 0.046, Figure 1B), the produced iMSCs displayed similar mtDNA copy numbers between the OA and normal groups (Figure 1B).

In addition to the quantitative assessment of heteroplasmic variant burden, allele frequencies and mtDNA copy number, we also examined the qualitative distribution of SNVs across the mitochondrial genome. We found that in primary MSC_OA, the majority (60.67%) of heteroplasmic variants were located within protein-coding regions, suggesting a potential direct effect on mitochondrial protein synthesis and OXPHOS function. The predominance of coding-region variants persisted, albeit at lower proportions, following reprogramming into iPSCs (45.78%) and subsequent differentiation into iMSCs (56%). Notably, we observed that at the iMSC stage, no variants were detected within tRNA genes, while a relative increase in variants mapped to regulatory non-coding regions, particularly the control region (CR). The proportion of variants localized in rRNA genes remained relatively constant across all OA-derived cell types, representing approximately 25% of the total heteroplasmic variants (Figure 1C). A comparable localization pattern was observed in all cell populations derived from healthy donors, although the overall number of heteroplasmic variants was markedly lower. Specifically, we found that in MSC_N, approximately 60% of variants were found in coding regions, while very few mapped to regulatory sequences. During reprogramming and redifferentiation, as the total heteroplasmic load further declined, no tRNA-associated variants were detected (Figure 1D).

### 3.2. Reprogramming Reduces Variant Burden in OXPHOS and Mitochondrial Ribosomal Genes in OA-Derived iMSCs

**Figure 2:**
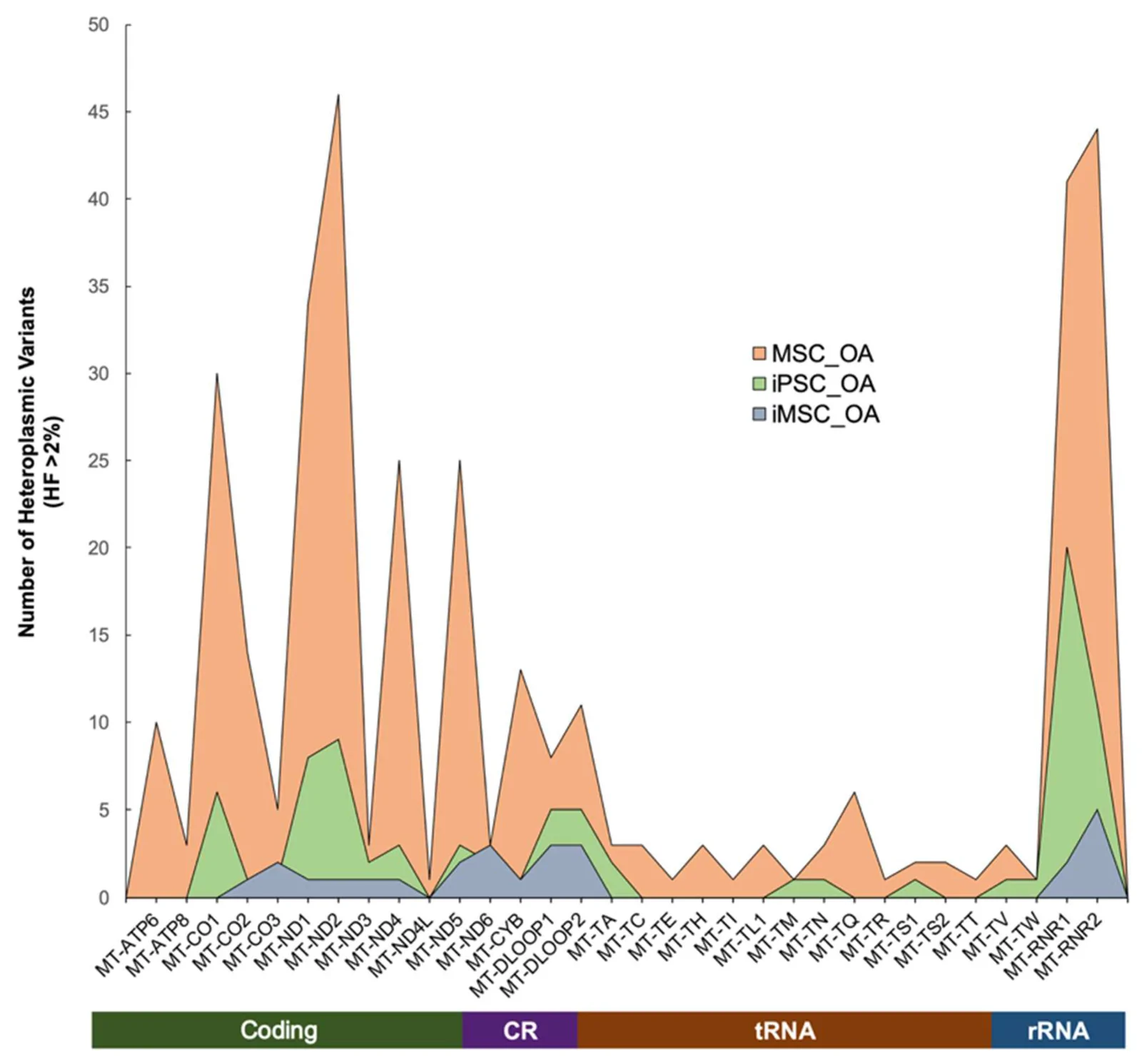
Bar plot illustrating the number of heteroplasmic SNVs detected per mitochondrial gene in OA-derived cell populations: MSC_OA (*n* = 3) (orange), iPSC_OA (*n* = 3) (green), and iMSC_OA (*n* = 3) (blue) (Supplementary Table S1).

We next sought to investigate the heteroplasmic SNV accumulation per mitochondrial gene in the OA-derived cell populations (MSCs, iPSCs, iMSCs). We observed that in MSC_OA, certain mitochondrial genes emerged as regions of SNV enrichment. Notably, MT-ND1, MT-ND2, MT-ND3 and MT-ND5 genes, which encode subunits of Complex I of the respiratory chain; MT-ATP8 encoding Complex V, an ATP synthase subunit; and MT-CO1 encoding Complex IV, a cytochrome c oxidase subunit 1, harbored, on average, approximately 30 heteroplasmic variants each (Figure 2). Following reprogramming of MSCs into iPSCs and their subsequent differentiation into iMSCs, a marked reduction in variant load to less than five variants per gene was observed across the above mentioned six genes (Friedman’s test, MT-ND1, adjusted-*p* = 0.027, MT-ND2, adjusted-*p* = 0.032, MT_ND3, adjusted-*p* = 0.0193, MT-ND5, adjusted-*p* = 0.0441, MT-ATP8, adjusted-*p* = 0.047, MT-CO1, adjusted-*p* = 0.036). A similar pattern was evident in the ribosomal RNA genes MT-RNR1 and MT-RNR2, which encode the 12S and 16S rRNAs, respectively. In primary MSC_OA, MT-RNR1 and MT-RNR2 exhibited particularly high variant counts (approximately 40 SNVs), while, following reprogramming and redifferentiation, nearly all rRNA variants were eliminated and approached the low counts observed in the two healthy-donor-derived cell lines (Friedman’s test, MT-RNR1, adjusted-*p* = 0.041, MT-RNR2, adjusted-*p* = 0.046, Figure 2). In samples derived from healthy donors, the distribution of mitochondrial variants across the different cell types did not demonstrate any distinct pattern. Overall variant burden remained low, and no recurrently altered regions or mutational “hotspots” were observed (Supplementary Figure S1).

### 3.3. Variants with Higher Predicted Pathogenicity Scores Are Reduced Across OA-Derived Cell States

**Figure 3:**
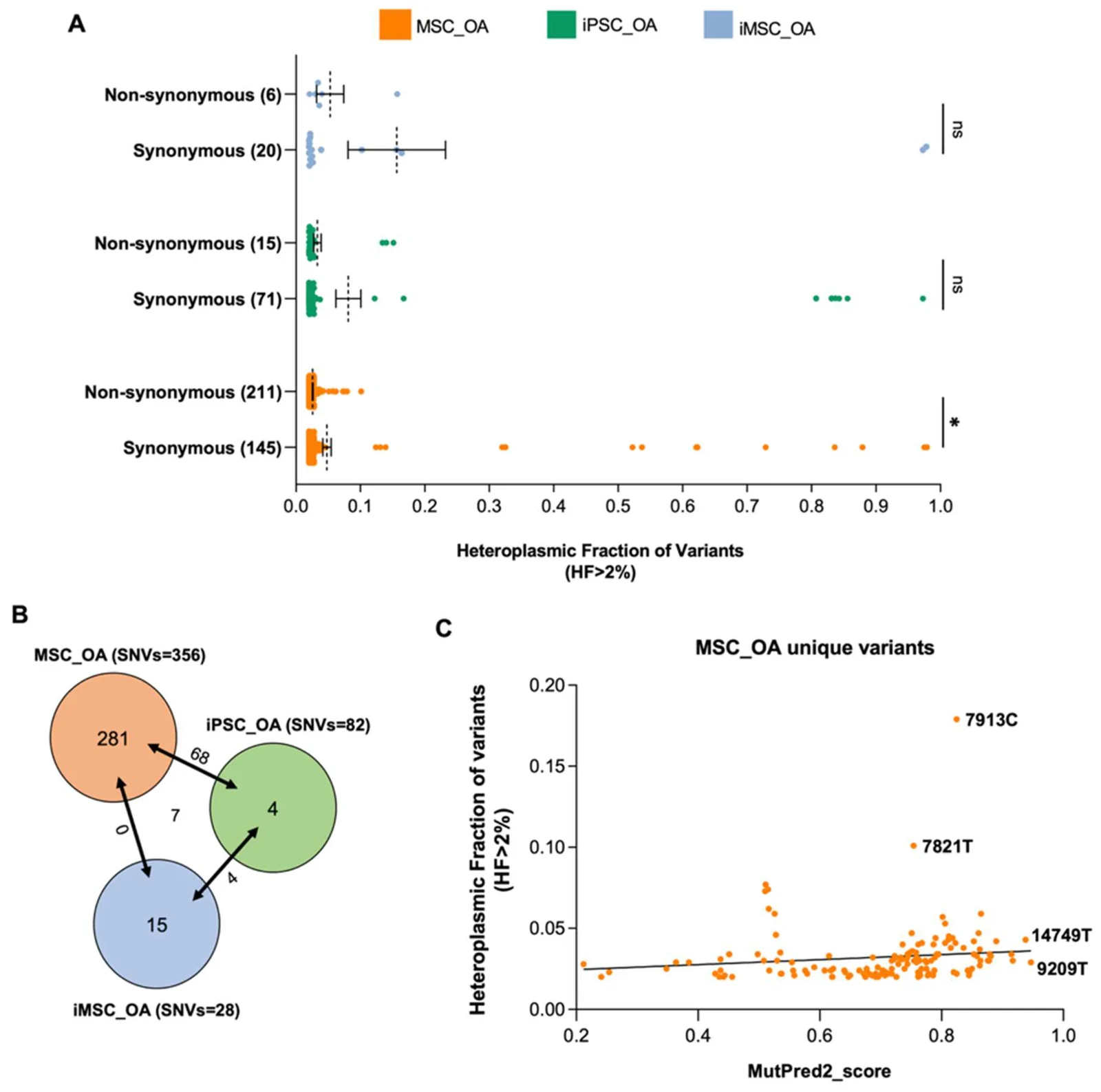
(**A**) Distribution of synonymous and non-synonymous SNVs across MSC_OA (*n* = 3) (orange), iPSC_OA (*n* = 3) (green), and iMSC_OA (*n* = 3) (blue). (**B**) Venn diagram showing unique and common variants among OA cell types. (**C**) Correlation between heteroplasmic fraction and MutPred pathogenicity score for unique variants in MSC_OA. Pearson’s r = 0.82, 95% CI [−0.042, 0.035], R^2^ = 0.017, * *p* = 0.0122; ns = no significance.

To assess the potential functional impact of the heteroplasmic variants identified in MSC_OA, the protein-coding SNVs were stratified into synonymous and non-synonymous (Figure 3A). Across all cell types, differences in variant composition were observed between synonymous and non-synonymous substitutions. In both iPSCs and iMSCs, synonymous variants outnumbered non-synonymous variants, indicating a relative predominance of functionally neutral substitutions. Analysis of heteroplasmic fractions (HFs) revealed no significant differences between variant classes in iPSCs and iMSCs, although synonymous variants tended to exhibit higher mean HFs (Mann–Whitney U-test, iPSCs *p* = 0.137; iMSCs *p* = 0.42). In contrast, within MSC_OA, non-synonymous variants were more abundant than synonymous variants (211 vs. 145), reflecting a shift toward protein-altering mitochondrial variation in the OA-derived MSC samples examined (HFs between synonymous and non-synonymous variants, Mann–Whitney U-test, *p* = 0.0431).

To further investigate the above observation, we analyzed the origin and persistence of each variant by comparing the complete SNV profile among all OA cell types (Figure 3B). Variants unique to each cellular population were subsequently assessed for the relationship between their heteroplasmic fraction and predicted pathogenic potential using the MutPred algorithm. We observed that among unique variants detected in MSC_OA (*n* of variants = 281, Supplementary Table S2), which were absent after reprogramming and redifferentiation, mutations with high MutPred scores (>0.6) indicative of elevated pathogenic potential tended to exhibit relatively high heteroplasmic fractions. Within the OA-derived MSC variant dataset, MutPred score showed a positive association with heteroplasmic fraction, implying that more potentially deleterious variants tended to accumulate at higher frequencies (Figure 3C). Although most variants displayed low heteroplasmy (<5%), two notable exceptions, m.7913C and m.7821T, were identified, which demonstrated relatively high heteroplasmy (~18%) and elevated MutPred score (~0.80).

Moreover, three additional variants, m.3668T, m.14749G, and m.4789T, displayed very high predicted pathogenicity (~0.95) despite low heteroplasmic fractions, suggesting potential functional impact even at subthreshold levels. In contrast, 15 unique variants (Supplementary Table S2) detected exclusively in iMSCs_OA were characterized by very low heteroplasmy (~2%) and minimal predicted pathogenicity, indicating limited biological significance. These variants may represent newly acquired variants or clone-specific expansion events during reprogramming and differentiation. Exceptions were the variants m.9939A and m.1399C which showed marginal MutPred scores but remained at low heteroplasmy levels, suggesting negligible functional effect (Supplementary Table S3).

### 3.4. Regulation of Nuclear-Encoded Mitochondrial Genes Reveals Metabolic Reprogramming During the MSC_OA to iMSC_OA Transition

**Figure 4:**
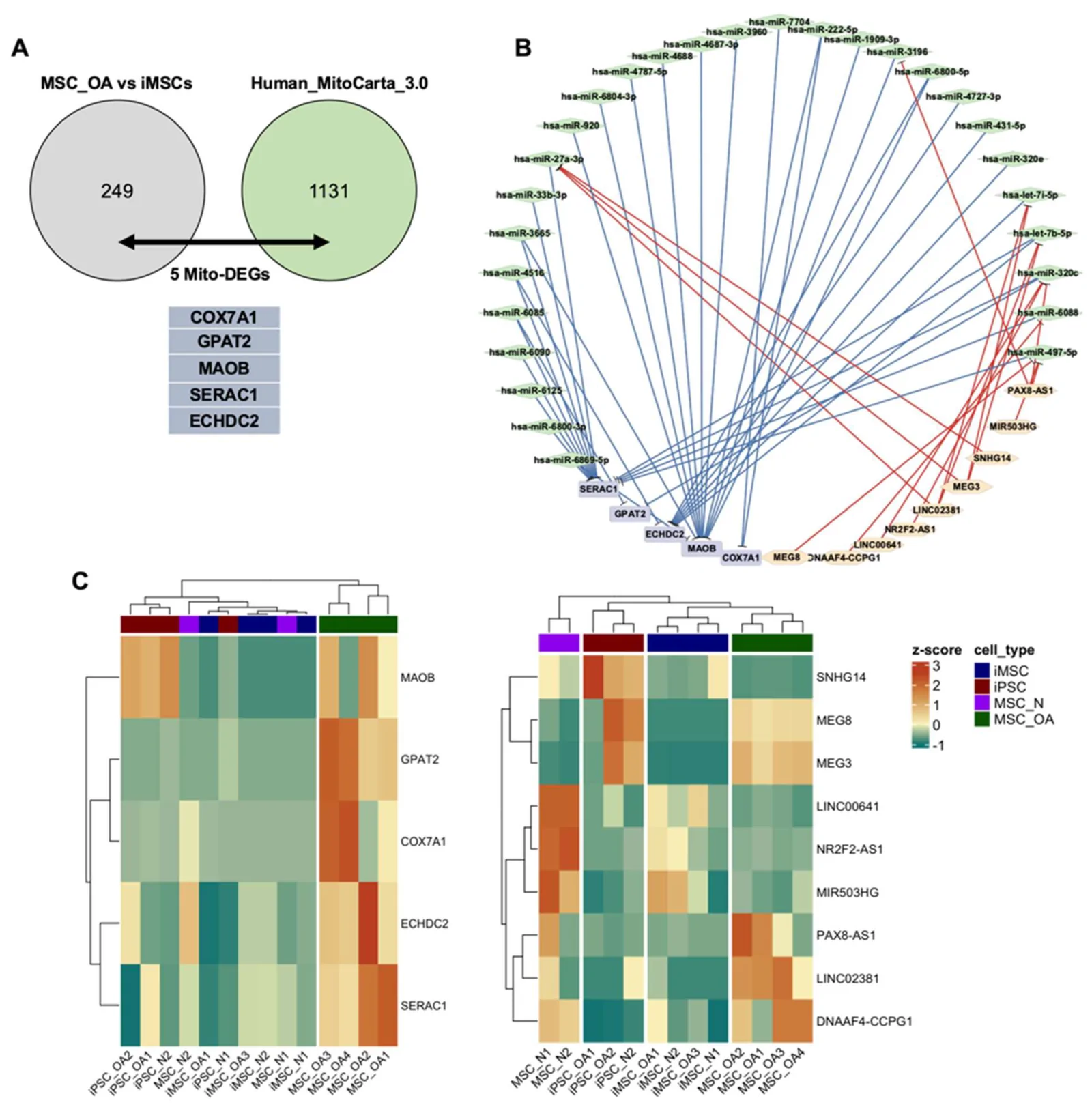
(**A**) Venn diagram showing the overlap between differentially expressed genes (DEGs) identified in the comparison between MSC_OA and iMSC_OA and the nuclear genes related to mitochondrial function (*COX7A1, GPAT2, MAOB, SERAC1* and *ECHDC2*) listed in the Human MitoCarta 3.0 database. (**B**) ceRNA network illustrating relationships between differentially expressed mitochondrial mRNAs, miRNAs and lncRNAs in iMSCs (lncRNAs = orange; miRNAs = green; mRNAs = purple; red lines indicate lncRNA-miRNA interactions; blue lines indicate miRNA-mRNA interactions). (**C**) Expression profiles of the five nuclear genes and nine hub lncRNAs functionally linked to mitochondrial activity in MSCs, iPSCs and iMSCs.

To identify nuclear-encoded genes functionally linked to mitochondrial activity, the previously identified by us differentially expressed genes (DEGs) in iMSCs [23] were cross-referenced with the MitoCarta 3.0 database. The comparison revealed five genes, *COX7A1, GPAT2, MAOB, SERAC1*, and *ECHDC2*, that were directly associated with mitochondrial function (Figure 4A). These five genes exhibited markedly reduced expression in iMSCs_OA, resembling that of iPSCs and MSCs derived from healthy donors (MSC_N) (Figure 4C).

We then constructed a lncRNA-miRNA-mRNA competing endogenous RNA (ceRNA) interaction network incorporating, exclusively, the identified nuclear genes and their interacting regulatory RNAs in OA-derived cell types (Figure 4B). Network analysis revealed complex interactions between 28 hub miRNAs, such as let7b/i, miR-497-5p, and 9 hub lncRNAs, such as MEG3/8, SNHG14, displaying inverse expression patterns. We observed that the iMSCs-lncRNA profile formed an intermediate state between iPSCs and MSC_OA, indicating partial reset of disease-associated post-transcriptional signatures following reprogramming.

## 4. Discussion

Recent evidence has revealed that mitochondrial dysfunction in aged cells, manifested as mtDNA damage, altered mitochondrial dynamics and metabolic imbalances, contributes to several diseases, such as cancer, neurodegenerative, cardiovascular, liver and metabolic diseases [29,30]. A significant role of mitochondrial dysfunction has also been attributed to the onset and progression of osteoarthritis [3].

In the present study, using ultra-deep sequencing, we evaluated single-nucleotide variants (SNVs) in MSCs, iPSCs, and iMSCs populations derived from healthy donors and OA patients. MSCs from OA patients (MSC_OA) revealed elevated heteroplasmy reflecting mitochondrial genomic instability associated with aging and chronic oxidative stress [31], conditions that are present in OA [32]. The observed increase in heteroplasmic SNVs counts is compatible with previous evidence indicating that aged MSCs harbor increased mtDNA SNV burden, particularly in genes associated with Complex I and Complex IV, contributing to reduced oxidative phosphorylation efficiency and impaired cellular energy output [12,33]. However, as age differed between the donor groups, the present study cannot determine whether differences observed between groups are attributable to OA, aging or their combined effects. Additionally, all three matched OA donor trajectories showed a reduction in heteroplasmic variant burden following reprogramming into iPSCs, which remained low after differentiation into iMSCs. This intra-donor pattern is consistent with mitochondrial genome remodeling during reprogramming. Nevertheless, the limited number of donors prevents determination of how consistently this pattern occurs across the universal OA patients. This observation is in accordance with recent studies reporting that iPSCs and iMSCs retain a remodeled mitochondrial genome with lower mutational burden accompanied by energetic recovery through reactivation of OXPHOS and improved ATP-production capacity [13,34]. Moreover, mtDNA copy number quantification during reprogramming revealed that OA-derived MSCs exhibited a lower mtDNA copy number than normal MSCs. Following reprogramming and subsequent differentiation, this difference was reduced, with iPSCs and iMSCs derived from both groups displaying more comparable mtDNA copy numbers [16]. This finding suggests that reprogramming partially increases mitochondrial content, potentially reflecting enhanced cellular energy production, although the underlying mechanisms require further investigation. Accordingly, the observed reduction in mtDNA variant burden and increased mtDNA copy number may represent a genomic component of mitochondrial adaptation during reprogramming. Whether this change results in improved mitochondrial quality or energy production remains to be established using functional assays.

Regarding qualitative characteristics in the mitochondrial genome, we found that in MSC_OA, certain mitochondrial genes, such as MTND1/2/3/5MT-ATP8 and MT-CO1 encoding subunits of complexes I, V, and IV of the oxidative phosphorylation (OXPHOS) system, emerged as distinct “hotspots” of heteroplasmic SNVs accumulation. The above novel finding is of particular interest, as these SNVs “hotspots” affect critical components of the OXPHOS machinery, potentially compromising mitochondrial energy production in MSCs derived from OA patients and contributing to cellular dysfunction in OA. Similar patterns have been reported in MSCs and chondrocytes isolated from OA tissues, where complex I dysfunction has been linked to increased reactive oxygen species (ROS) generation, disruption of mitochondrial membrane dynamics, and accelerated cellular senescence [35,36,37]. Following MSC_OA differentiation into iPSC_OA and subsequently into iMSC_OA, we observed elimination of variants localized to “SNVs hotspots”. The observed reduction in SNVs in MT-ND, MT-CO, and MT-ATP8 genes, as well as in MT-RNR1 and MT-RNR2 encoding the 12S and 16S rRNAs, is suggestive of a partial “genetic reset” of the mitochondrial genome in iPSCs and iMSCs. These observations are consistent with the hypothesis that cells harboring high-heteroplasmy pathogenic mtDNA variants may be subject to negative (purifying) selection during the acquisition of pluripotency and lineage-specific differentiation, as previously proposed [38]. However, the present study did not directly investigate the mechanisms underlying this process or its effects on mitochondrial function or reprogramming efficiency.

Interestingly, despite the increased number of SNVs observed in primary MSC_OA, the heteroplasmic fraction of most variants remained low (2–5%), likely indicating a polyclonal mitochondrial population, in which multiple low-frequency mtDNA genotypes coexist without a single dominant genome being positively selected [39]. In all reprogrammed cell states, iPSCs and iMSCs of healthy donors, we observed that synonymous variants exhibited higher heteroplasmic fractions consistent with neutral drift, rather than selective pressure. In contrast, in MSC_OA, an enrichment of non-synonymous variants was observed, which allows potentially deleterious substitutions to persist and expand within the mitochondrial population. This pattern, which is in line with the chronic oxidative and inflammatory environment of OA, may reflect disrupted mitochondrial surveillance mechanisms, including mitophagy, representing a cellular response to chronic damage and aging.

Based on the above findings, we then proceeded to identify non-synonymous mtDNA variants unique to MSC_OA and evaluate their potential functional impact. We identified a subset of variants (*n* = 281) exclusive to MSC_OA that were no longer detectable following reprogramming. Notably, variants with higher predicted pathogenicity, as indicated by elevated MutPred scores (>0.6), were associated with relatively increased heteroplasmic fractions. Furthermore, the positive correlation observed between heteroplasmic fraction and predicted pathogenic potential among MSC_OA-specific variants suggests that potentially deleterious substitutions can accumulate to appreciable levels in the OA cellular state, suggesting altered maintenance or segregation of mtDNA variants prior to reprogramming [40,41]. Notably, the identification of two rare, potentially pathogenic according to MITOMAP [42] and the literature [43,44], MT-CO2 variants, m.7821C>T and m.7913T>C, with elevated MutPred scores and relatively high heteroplasmic fraction underscores the potential for direct impairment of complex IV activity with downstream consequences for ATP production and ROS generation. However, the observed persistence in MSC_OA cells of the additional predicted to be pathogenic variants, m.3668T, m.14749G, and m.4789T, at low heteroplasmic fractions suggests that even low-threshold mtDNA alterations may contribute cumulatively to mitochondrial dysfunction in OA-derived MSCs [12]. Importantly, the complete absence of high-heteroplasmy pathogenic variants in iPSC_OA and iMSC_OA is consistent with, but does not demonstrate, the operation of purifying selection during reprogramming and redifferentiation. This interpretation, therefore, remains hypothetical.

In addition, mtDNA remodeling should not be interpreted only as the elimination of pre-existing variants. Cellular reprogramming may also lead to the emergence of detectable mtDNA variants through mitochondrial bottleneck effects, clonal expansion or de novo mutational events. As a result, the variants detected exclusively in iMSCs may represent either newly acquired mutations or the expansion of pre-existing low-level heteroplasmic variants that were below the detection threshold in parental cells during reprogramming and subsequent differentiation.

Taking into consideration that mitochondrial function is not only regulated by the mitochondrial genome, but nuclear-encoded genes, also, play critical roles in mitochondrial functionality, we proceeded, for the first time to our knowledge, to examine the behavior of the nuclear-encoded mitochondrial-associated genes. Cross-reference of the previously found by us differentially expressed genes (DEGs) in iMSCs during the differentiation process from MSCs_OA to iMSCs [23] with nuclear genes encoding mitochondrial proteins from the MitoCarta database [28], revealed five genes, *COX7A1*, *GPAT2*, *MAOB*, *SERAC1*, and *ECHDC2*, that were associated with mitochondrial function. All five nuclear genes exhibited reduced expression in iMSCs adopting an expression profile that closely resembled that of iPSCs and MSCs derived from healthy donors rather than MSC_OA samples. The observed reduced expression of *COX7A1* and *MAOB* in iMSCs may indicate an adaptive down-tuning of mitochondrial oxidative activity to avoid excess ROS generation, a profile compatible with post-rejuvenation metabolic stabilization state [45,46]. *COX7A1*, a subunit of Complex IV, has been linked to oxidative stress responses in aged or inflammatory cells [47,48], while *MAOB* inhibition has been associated with mitochondrial protection and improved stem cell energetic output [49,50]. In parallel, genes such as *SERAC1*, *GPAT2*, and *ECHDC2*, which regulate the phospholipid recycling and the architecture of the inner mitochondrial membrane system [51], have established roles in mitochondrial membrane integrity and OXPHOS efficiency. *SERAC1* is essential for remodeling phosphatidylglycerol species and cardiolipin, a key structural component of the mitochondrial membrane whose dysfunction is associated with impaired mitochondrial biogenesis [52]. GPAT2, a regulator of lipid homeostasis, has been reported to have reduced expression in primary MSC populations [53]. The observed suppression of *GPAT2* in iMSCs may reflect metabolic rebalancing following removal of OA-associated oxidative stress. In that regard, the combined downregulation of the nuclear-encoded mitochondrial-related genes may, therefore, indicate an adjustment in lipid-biosynthetic mechanisms to restore mitochondrial membrane stability and reduce metabolic fragility.

Finally, the constructed lncRNA–miRNA–mRNA interaction networks for *COX7A1*, *GPAT2*, *MAOB*, *SERAC1*, and *ECHDC2* revealed a complex post-transcriptional regulatory framework. Multiple lncRNAs, such as MEG3 and SNHG14, may function as competing endogenous RNA (ceRNA) “sponges” targeting miRNAs [54,55], including hsa-miR-145, hsa-miR-195 and hsa-let-7b family members, ultimately modulating their gene targets. Integration of these multiomic layers suggests that post-transcriptional RNA interactions may be associated with mitochondrial-related molecular changes in iMSCs. Whether these regulatory changes improve energetic efficiency or reduce metabolic stress remains to be determined by functional studies [56]. The present findings further suggest that the observed reduction in mtDNA variants in iMSCs occurs in parallel with changes in the expression of nuclear-encoded mitochondrial genes and regulatory RNAs. These observations indicate coordinated transcriptional and post-transcriptional changes during reprogramming but do not establish a causal relationship between these molecular changes and mitochondrial genome remodeling. Likewise, they do not directly demonstrate restoration of mitochondrial function or energetic efficiency.

Several limitations should be considered when interpreting these findings. First, the study included three OA donors and two healthy donors, limiting the statistical power and generalizability of the findings. Although the matched MSC-iPSC-iMSC design allowed each donor to serve as its own biological reference for assessing mitochondrial genomic changes during cellular transitions, it does not capture the full extent of inter individual mtDNA heterogeneity. Furthermore, the observed patterns should be regarded as exploratory and hypothesis-generating rather than evidence of universal biological mechanisms. Finally, validation in larger, independent, age-matched donor cohorts, together with direct functional analyses of mitochondrial physiology, including measurements of oxygen consumption rate (OCR), ATP production, mitochondrial membrane potential and mitophagy markers, will be necessary to establish the reproducibility and biological significance of these observations.

Collectively, this exploratory study provides novel insights into mitochondrial genome remodeling during cellular reprogramming in donor-derived MSCs, iPSCs and iMSCs. The reduction in mtDNA variants observed in the analyzed donor-derived cell lines warrants further investigation of mitochondrial genome remodeling during cellular reprogramming and differentiation, as well as the mechanisms underlying these changes. The present findings also support the utility of reprogrammed cell models for investigating mitochondrial genomic dynamics in OA. Furthermore, the potential applicability of these findings in regenerative medicine remains to be established through comprehensive functional, safety, and in vivo studies.

## Data Availability

Data are contained within the article and Supplementary Materials. Data are available upon request to the corresponding author.

## Supplementary Materials

The following supporting information can be downloaded at: https://www.mdpi.com/article/10.3390/genes17091101/s1, Table S1: Mitochondrial genes and relation to OA mitochondrial dysfunction; Table S2: Unique variants detected in MSC_OA with high MutPred scores (>0.6) and high heteroplasmic fraction (HF>0.2); Table S3: Variants detected exclusively in iMSCs_OA were characterized by very low heteroplasmic fraction (~2%) and minimal predicted pathogenicity score; Figure S1: Bar plot illustrating the number of heteroplasmic SNVs detected per mitochondrial gene in H-derived cell populations.

## Abbreviations

7-AAD, 7-aminoactinomycin D; AF, allele fraction; BM, bone marrow; ceRNA, competing endogenous RNA; CR, control region; DEGs, differentially expressed genes; FC, fold change; FDR, false discovery rate; HF, heteroplasmic fraction; iMSCs, iPSC-derived mesenchymal stromal cells; iPSCs, induced pluripotent stem cells; MSC_N, mesenchymal stromal cells derived from healthy donors; MSC_OA, mesenchymal stromal cells derived from osteoarthritis patients; MSCs, mesenchymal stromal cells; mtDNA, mitochondrial DNA; mtSNVs, mitochondrial single-nucleotide variants; NUMTs, nuclear mitochondrial DNA segments; OA, osteoarthritis; OCR, oxygen consumption rate; OXPHOS, oxidative phosphorylation; rCRS, revised Cambridge Reference Sequence; RNA, ribonucleic acid; ROS, reactive oxygen species; SNVs, single-nucleotide variants.
