# Supplementary material for: Characterization of the Mitochondrial Genome Landscape in MSCs, iPSCs and iMSCs from Osteoarthritis Patients and Healthy Donors
Source: Genes (Basel). 2026 Sep 11;17(9):1101. doi: 10.3390/genes17091101 (PMC13606117; doi:10.3390/genes17091101)
Supplement: Supplementary file 1 [file genes-17-01101-s001.zip › Supplementary_Table_S1.pdf]

Gene	Encoded Protein/ OXPHOS Role	OA-related mitochondrial dysfunction
MT-ND1, MT-ND2, MT-ND3, MT-ND5	Complex I subunits/ Electron transport and proton pumping	Affect respiratory efficiency and ROS production
MT-CO1	Complex IV subunit/ Terminal electron transfer and oxygen reduction	Impair respiration and ATP generation
MT-ATP8	ATP synthase subunit 8/ ATP synthesis and Complex V assembly	Influence cellular energy production
MT-RNR1	12S mitochondrial rRNA/ Mitochondrial ribosome function	Altered translation may affect synthesis of OXPHOS proteins
MT-RNR2	16S mitochondrial rRNA/ Mitochondrial ribosome function.	Variants may influence mitochondrial protein synthesis and respiratory-chain integrity

Supplementary Table S1: Mitochondrial genes and relation to OA mitochondrial dysfunction
