# Supplementary material for: Characterization of the Mitochondrial Genome Landscape in MSCs, iPSCs and iMSCs from Osteoarthritis Patients and Healthy Donors
Source: Genes (Basel). 2026 Sep 11;17(9):1101. doi: 10.3390/genes17091101 (PMC13606117; doi:10.3390/genes17091101)
Supplement: Supplementary file 1 [file genes-17-01101-s001.zip › Supplementary_Table_S2.pdf]

MSC_OA unique SNVs	Pathogenicity Score (MutPred Score)	Heteroplasmic Fraction (HF)
7384T	0.779	0.022
4121T	0.783	0.03
9253T	0.786	0.021
10182T	0.787	0.04
10375T	0.788	0.024
7760A	0.789	0.032
10830T	0.79	0.044
4127T	0.792	0.034
6211T	0.795	0.034
3882T	0.802	0.057
12818T	0.806	0.053
6904T	0.806	0.041
8648T	0.811	0.043
5035T	0.812	0.045
3707T	0.815	0.038
8168A	0.818	0.044
4632A	0.82	0.023
14976T	0.823	0.041
4585A	0.825	0.022
7913C	0.825	0.179
11777A	0.836	0.038
6808T	0.843	0.024
6537T	0.845	0.021
4037T	0.846	0.023
3890T	0.85	0.025
10917A	0.853	0.034
5014A	0.853	0.042
4720T	0.858	0.033
5413T	0.861	0.047
11403T	0.863	0.037
6280T	0.863	0.027
5170T	0.864	0.03
7008T	0.865	0.059
8073T	0.876	0.03
7252T	0.878	0.03
4153T	0.879	0.034
13225T	0.881	0.033
11592T	0.89	0.042
3668T	0.915	0.034
4789T	0.917	0.03
14749T	0.938	0.043
9209T	0.947	0.029

Supplementary Table S2. Unique variants detected in MSC_OA with high MutPred scores (>0.6) and high heteroplasmic fraction (HF>0.2)
