# Supplementary material for: Characterization of the Mitochondrial Genome Landscape in MSCs, iPSCs and iMSCs from Osteoarthritis Patients and Healthy Donors
Source: Genes (Basel). 2026 Sep 11;17(9):1101. doi: 10.3390/genes17091101 (PMC13606117; doi:10.3390/genes17091101)
Supplement: Supplementary file 1 [file genes-17-01101-s001.zip › Supplementary_Table_S3.pdf]

iMSC_OA unique SNVs	Pathogenicity Score (MutPred Score)	Heteroplasmic Fraction (HF)
13369C	0.83	0.03
14530C	0	0.025
152C	0	0.97
15758G	0.049	0.034
16289G	0	0.026
16304C	0	0.97
16519C	0	0.022
195C	0	0.021
2623G	0	0.039
3003G	0.3	0.02
3919C	0.2	0.04
4136G	0.37	0.164
8104G	0.83	0.036
9923T	0	0.021
9939A	0.7	0.04

Supplementary Table S3. Variants detected exclusively in iMSCs_OA were characterized by very low heteroplasmic fraction (~2%) and minimal predicted pathogenicity score
